# Effect of Infant Presence on Social Networks of Sterilized and Intact Wild Female Balinese Macaques (*Macaca fascicularis*)

**DOI:** 10.3390/ani11092538

**Published:** 2021-08-29

**Authors:** Gwennan Giraud, Sebastian Sosa, Alain Hambuckers, Stefan Deleuze, I Nengah Wandia, Marie-Claude Huynen, Pascal Poncin, Fany Brotcorne

**Affiliations:** 1 Research Unit SPHERES, Department of Biology, Ecology and Evolution, Faculty of Sciences, University of Liège, 4020 Liège, Belgium; alain.hambuckers@uliege.be (A.H.); Marie-Claude.Huynen@uliege.be (M.-C.H.); fbrotcorne@uliege.be (F.B.); 2 Department of Ecology, Physiology and Ethology, University of Strasbourg, CNRS, IPHC UMR 7178, 67200 Strasbourg, France; s.sosa@live.fr; 3 Research Unit FARAH, Equine and Companion Animal Reproduction Pathologies Clinic, Faculty of Veterinary Medicine, University of Liège, Sart-Tilman, 4130 Liège, Belgium; s.deleuze@uliege.be; 4 Primate Division of Natural Resources and Environment Research Center, Faculty of Veterinary Medicine, Universitas Udayana, Denpasar 80361, Bali, Indonesia; wandia@unud.ac.id; 5 Research Unit FOCUS, Department of Biology, Ecology and Evolution, Faculty of Sciences, University of Liège, 4020 Liège, Belgium; P.Poncin@uliege.be

**Keywords:** birth control, ego network, infant attraction, reproductive condition, social position, social role, tubectomy, urban macaques, welfare

## Abstract

**Simple Summary:**

In primates, social interactions are significantly influenced by the female reproductive cycle and the presence of infants. In particular, unweaned infants act as amplifiers of social interactions: they facilitate contact between group members while also triggering occasional conflicts. Sterilized females no longer having young offspring could see their social integration modified. We studied intact and recently sterilized wild female long-tailed macaques (*Macaca fascicularis*) to see if infant presence was influencing the females’ place and role in the social group. We used the social network analysis tool to compare grooming and proximity to other females for females in three nursing conditions (with young infant (YI), with old infant (OI), and non-nursing (NN). YI females were less involved in grooming but stayed in closer proximity to other females than OI and NN females. We suggest that YI females keeping proximity to others was a way to maximize their infants’ protection, while avoiding too direct social interactions, such as grooming, to protect them from kidnapping risk. Overall, sterilization did not deteriorate female social integration, at least shortly after the surgery. Further research should track the social status of non-nursing females over a long time period, in aid of making sound population management decisions.

**Abstract:**

Contraception is increasingly used to control wild animal populations. However, as reproductive condition influences social interactions in primates, the absence of new offspring could influence the females’ social integration. We studied two groups of wild macaques (*Macaca fascicularis*) including females recently sterilized in the Ubud Monkey Forest, Indonesia. We used social network analysis to examine female grooming and proximity networks and investigated the role of infant presence on social centrality and group connectivity, while controlling for the fertility status (sterilized N = 14, intact N = 34). We compared the ego networks of females experiencing different nursing conditions (young infant (YI) vs. old infant (OI) vs. non-nursing (NN) females). YI females were less central in the grooming network than other females while being more central in proximity networks, suggesting they could keep proximity within the group to protect their infant from hazards, while decreasing direct grooming interactions, involving potential risks such as kidnapping. The centrality of sterilized and intact females was similar, except for the proximity network where sterilized females had more partners and a better group connectivity. These results confirm the influence of nursing condition in female macaque social networks and did not show any negative short-term effects of sterilization on social integration.

## 1. Introduction

In social mammals, reproductive status and the mere presence of infants may influence social associations and interactions, those conditions being largely under hormonal processes [1,2,3]. Natal attraction and allo-parental behaviors are well spread in social species [4,5,6,7], especially in K-selected mammal species with low reproductive rates [8], allowing partners to socialize, which is particularly important for individual fitness [9,10]. In cercopithecine primates, unweaned infants are mediators and amplifiers of female social ties since they play a role in the biological market [11,12]. Studies have shown that attraction to infants and its influence on social bonds are species-specific. For example, within the macaque genus, females of different species are highly to moderately permissive with their infants and more or less tolerant toward females approaching them, particularly during the first month of the infant’s life [13,14]. Infant handling by group members is frequent and attraction for newborns is particularly significant for matrilineal primates such as macaques and baboons where mothers are often the focus of other females’ attention [6,7,15,16].

Whereas, former studies on this topic have been conducted at a dyadic level [6,7,11,13,15,17,18], some authors have suggested that infant presence might have a greater influence than expected at the group level [2,16]. In capuchins (*Sapajus* spp.) for example, infants improve the centrality of nursing females in the grooming network without enhancing their spatial or hierarchical positions [19]. Therefore, in nepotistic social systems such as macaques, infants and young offspring might play a key role in the mother’s centrality [19] and may play an extensive role in group social cohesion [20,21] if we assume that grooming a mother is a way to access young infants [11]. To shed light on this realm, social network analysis (SNA) is an efficient tool allowing us to depict relationships at the individual, group and population levels, and to understand the fitness implications of social relationships [22,23,24,25,26,27]. The group level allows for the differentiation between the social position and social role. The social role refers to the way an individual position influences or is influenced by the position of other social partners [28,29]. For example, it has been shown in baboons that juveniles contribute to the social structure of the overall group and influence the social role of subadult and adult females [21]. If dyadic level studies under-represent the social position and role of an individual within its group, the larger and more integrative picture provided by SNA tools allows us to deepen the study of social interaction patterns, the social position of individuals and their social roles within a group, and in turn, how the latter influences the former. SNA can focus on both ego network (the individual ‘ego’ as the actor of their own network [30]) and the individual position within the group, and is thus a perfect tool to assess changes in individuals’ direct and indirect social interactions related to specific factors. Therefore, SNA might help to test the hypothesis whereby infants play a crucial role in building and strengthening adult females’ social bonds, by analyzing the relationship between the presence of unweaned infants and the female’s centrality (social position) and connectivity (social role) within the network.

Sociality and welfare are closely linked in social species [31,32]. While animal welfare has been deeply investigated in farm and captive settings, studies in wild populations remain sparse [31,33]. It is particularly true in human-modified environments where people attempt to manage wild populations [33]. Changes induced by management strategies, such as birth control to control wildlife population growth, might impose additional costs to individuals’ social life and welfare [34]. Welfare is quantified through individual physiological and behavioral indicators but also through group-level related measures since individuals and the group influence each other [28,31,35]. The effect of sociality on individual fitness emphasizes the importance of studying potential changes in sociality related to animal sterilization and therefore the implications of wildlife management on welfare.

In some regions, wildlife sterilization is increasingly used [36,37,38,39,40,41,42,43], therefore modifying individual reproductive output, natality rate and ultimately demographic structure of the populations where the social life develops. So far, the possible impact of reproductive control on the social dynamics of free-ranging primates has been poorly investigated and deserves further attention [44]. Vasectomy and tubectomy keep intact the steroid hormonal functions underpinning normal sexual activity, while impeding fecundation [45,46,47,48]. However, the behavioral implications of these techniques in wild primates remain poorly explored. SNA can be used to predict or assess the effects of birth control on social network structure and social dynamics [22], and to understand the short and long-term implications of sterilization on individual fitness and sociality in primates.

The purpose of this analysis was to assess, using social network analysis, the potential role of unweaned infants on female social dynamics in two macaque groups recently subjected to a birth control program using fallopian tube ligation (tubectomy) in the Ubud Monkey Forest, Bali, Indonesia [43]. This paper investigated the short-term implications of sterilization since most of the studied sterilized females (12/14) were neutered since less than one year. How the absence of an unweaned infant might impact the sterilized females is particularly important to understand how birth control programs influence primate social dynamics. Considering that unweaned infants are particularly attractive to the mother’s female partners [6,7,15,16], we expected that females nursing a young infant (YI) or an old infant (OI) would be more central and better connected in the affiliative networks than the non-nursing females (NN). This hypothesis means that the YI and the OI females would have a higher number of female neighbors, a stronger weight of connections through frequency or time duration spent interacting or associating, and a higher group connectivity power in comparison to NN females, and that this effect could vary with the age of the infant. In chacma baboons (*Papio ursinus*) and rhesus macaques (*Macaca mulatta*), YI females are attracted to other YI females, but this attraction declines with the infant age [6,18]. We therefore expected a gradient related to infant age: YI females would be in the most central position in the group, this centrality decreasing as the infant grows older, to end in a less central position when the infant is weaned, i.e., in NN females. Similarly, we predicted that YI females would play a central role in the connectivity of the female network (high quality of social partners themselves well connected), followed by OI females and ultimately by NN females. Considering the fertile status of the females, most of the sterilized females in our study were treated from less than one year (<1 year N = 12; >1 year N = 2) and some of them were still nursing an infant during a part of the study (N = 2). Knowing the average 1.1 years of birth interval in this species [49], we therefore expected no negative short-term influence of the sterilized status on the centrality and connectivity power of the females, particularly considering that no hormonal disfunction was expected following the tubectomy procedure [45,47]. This study aimed to preliminarily assess the general influence of nursing condition and the potential short-term impact of sterilization on macaque social dynamics in order to enlighten the implications of sterilizations and strengthen our capacity to design high-quality management strategies promoting welfare in wild primate populations.

## 2. Materials and Methods

### 2.1. Study Site and Study Groups

The Ubud Monkey Forest (8°31′ S, 155°15′ E) is a touristic forest sanctuary located in central Bali, Indonesia, where wild long-tailed macaques (*Macaca fascicularis*) have been living commensally with people for decades [50]. Monkeys are provisioned daily with fruits and vegetables by stakeholders [51,52], and there is only a low predation pressure left. The study site is densely populated with 54 individuals per hectare (Appendix A), for a total population of 1099 divided into eight social groups in 2020. Between 2017 and 2020, the average annual growth of this population was 12%, the average birth interval was 1.2 years with an average birth rate of 0.53. A three-year sterilization program using female endoscopic tubectomy was launched in 2017 to manage the macaque population growth and allowed the sterilization of 136 females in the population [43]. The present study was conducted on two of the eight social groups, called Michelin & Utara hereafter, for which we had the most detailed dataset. In March 2020, Michelin group counted 136 individuals in total, while Utara group counted 33 individuals (see Appendix A for the demographic composition of the study groups). However, the social data included in this study concern female network only, including 28 intact females and 13 sterilized females in Michelin, and 6 intact females and one sterilized female in Utara (Appendix B). All studied females were sexually mature thus likely to attempt to breed, and most of them experienced different nursing conditions over the course of the study (Appendix B).

### 2.2. Data Collection

Data were collected from August to September 2019 and from December 2019 to March 2020 by two observers, G. G. and a field assistant with a 90% inter-observer reliability [53]. The macaques were fully habituated to human presence and the visibility was excellent (2–10 m).

#### 2.2.1. Determination of the Nursing Condition

As reproduction in this population is non-seasonal [54], female subjects experienced different nursing conditions over the study period. With a pregnancy period of approximatively 5.5 months in this species [55], we assessed the female nursing condition (NN, YI or OI) based on the date of birth of the female’s last infant. Infants were considered as weaned after one year when they ingested solid food and were competently independent during travelling and foraging [56]. Females–including intact and sterilized ones–who did not care for an unweaned infant were categorized as non-nursing females (NN). Except for infants born during the study, the exact age of infants were not known. As a result, we used the infant coat color change to define age class and thus female nursing condition: young infant (YI) with black-grey coat until ~5 months of age, and old infant (OI) with beige-brown coat until 1 year old [57]. With regard to the fertility status (sterilized vs. intact), this study started after the end of the sterilization campaign. However, most of the sterilized female subjects were contracepted within the previous year (N = 12; 86% of the sterilized females) and some were still nursing at some point of the study (N = 2) (see Appendix B for details of the study subjects).

#### 2.2.2. Behavioral Data

We used 15-min focal sampling [58], with the Animal Behaviour Pro V.1.2. app [59] to collect a total of 202 h on the 41 females of the Michelin group (mean of 4.9 h/ind.) and 30 h of focal observation on the 7 females of the Utara group (mean of 4.3 h/ind.). Focal samples were done semi-randomly, i.e., based on the first-seen individual, independently from its activity, while giving priority to individuals having the lowest cumulative data. We collected duration and direction of grooming interactions as well as the identity of the focal interactor(s). To test our hypotheses related to nursing condition while controlling for the confounding effect of hierarchical rank [3,60,61,62], we calculated the rank on weighted matrices generated from unidirectional agonistic interactions (cf. “winner/looser”) with the modified David’s’ score [63,64] using the steepness R package [65]. We then standardized the hierarchical rank with the group size.

We supplemented focal sampling with scan samples [58] every 5 min to collect association data (frequencies) on contact- and ≤5 m proximity neighbors. Proximity scans done in food provisioning areas were excluded from the dataset to limit bias related to food competition. We got a total of 3099 proximity scans in Michelin group (mean of 76 scans/ind.) and 492 proximity scans in Utara group (mean of 70 scans/ind.).

#### 2.2.3. Social Network Data

To build the female proximity and grooming networks, we compiled monthly undirected social matrices based on proximity frequencies, and directed social matrices based on duration of grooming interactions, respectively. We removed from the matrices all the non-focal individuals [66] and focal individuals who disappeared over the study. To take into account variation in sampling effort between focal individuals, we weighted the matrices with the total observation duration of each individual following Farine and Whitehead [26]. From the monthly matrices, we constructed the ego matrices considering only interactions with direct female social partners or proximity associations within 5 m around the ego individual during the whole study period [30] for each focal individual according to its nursing condition. Therefore, each focal female had as many ego matrices as different nursing conditions she experienced during the study (Appendix B).

### 2.3. Social Network Analysis

We focused on ego networks which show the local connections of an individual over time and how they differ to other ones [30]. We used several metrics to analyze the centrality and connectivity of focal subjects in the female grooming and proximity networks. At the individual level (the node), we used node’s direct interactions, meaning one step of distance from the ego node, such as the degree (number of social partners who interacted with the ego individual) [67] and the strength (the values–or number of interactions–of the node ties) [68] to weigh the relationships, and we considered the directionality when necessary (in- and out- degree/strength). Between local (individual) and global (group) characterization, we used node’s direct and indirect interactions through Laplacian centrality metric as an intermediate measure of a node centrality. This metric considers two steps of distance from the ego node, and thus attests of the role of the ego node in the group connectivity and cohesion by its suppression from the network [69]. To process the matrices, calculate and analyze the metrics, we used the R package *ANTs* [70] and we drew sociograms with NETDRAW, the graphic interface of UCINET 6 v. 6.678 [71].

#### Statistical Analysis

To test the influence of the nursing condition (YI vs. OI vs. NN females) and the fertility status (sterilized vs. intact) on individual centrality and group connectivity in the grooming and proximity networks, we ran separate generalized linear models (GLMMs) for each network metric as response variable (binomial family for degree and in-/out-degree transformed into proportions to control for the group size; Gaussian family for strength, in-/out-strength, and Laplacian centrality). For model construction, we included nursing condition as the main fixed predictor, fertility status, group size, hierarchical rank and their interactions as controlled fixed factors, and the group membership and the identity of individuals as random effects to account for the within-subject repeated observations. Likelihood ratio tests (LRT) were then used to compare the model including the various combination of effects, which allowed us to exclude non-significant effects (i.e., all interactions) and to limit the number of random effects to a minimum (group membership was removed). For the grooming network, we kept all the remaining fixed predictors (nursing condition, fertility status, rank, group size) in the models of in-strength, out-strength, and Laplacian centrality. However, we removed the hierarchical rank from the in-strength model because the deviance was more adapted without this predictor, although only marginally significant (LRT: deviance = −325.93, Df = 4, *p* = 0.06). We also compared the final models to their corresponding null models to test the joint significance of the remaining predictors.

All statistics were performed using R 4.0.3. software [72] and we checked for assumptions of model residual normality using Kolmogorov-Smirnov’s test. When the residual normality of the model was not respected (i.e., with Laplacian centrality and Strength metrics), we used the square root transformation. As inferential statistical techniques request, observations must be independent [28,73]. Since interaction data between same-group members broke this rule, we used the *ANTs* package [70] to permute our social networks prior to run GLMMs with a confidence interval set at 95%, as recommended by Whitehead [74] and Croft et al. [75] to obtain unbiased significance tests for the coefficients. More specifically, we used node label permutations (N = 10,000) on the metrics (argument ‘labels’ in function ‘perm.net.nl’) calculated from social matrices [76]. We ran Tukey post-hoc test using *lsmeans* package [77] to perform pairwise comparisons between nursing conditions.

## 3. Results

### 3.1. Effect of Nursing Condition on Proximity and Grooming Networks

**Figure 1:**
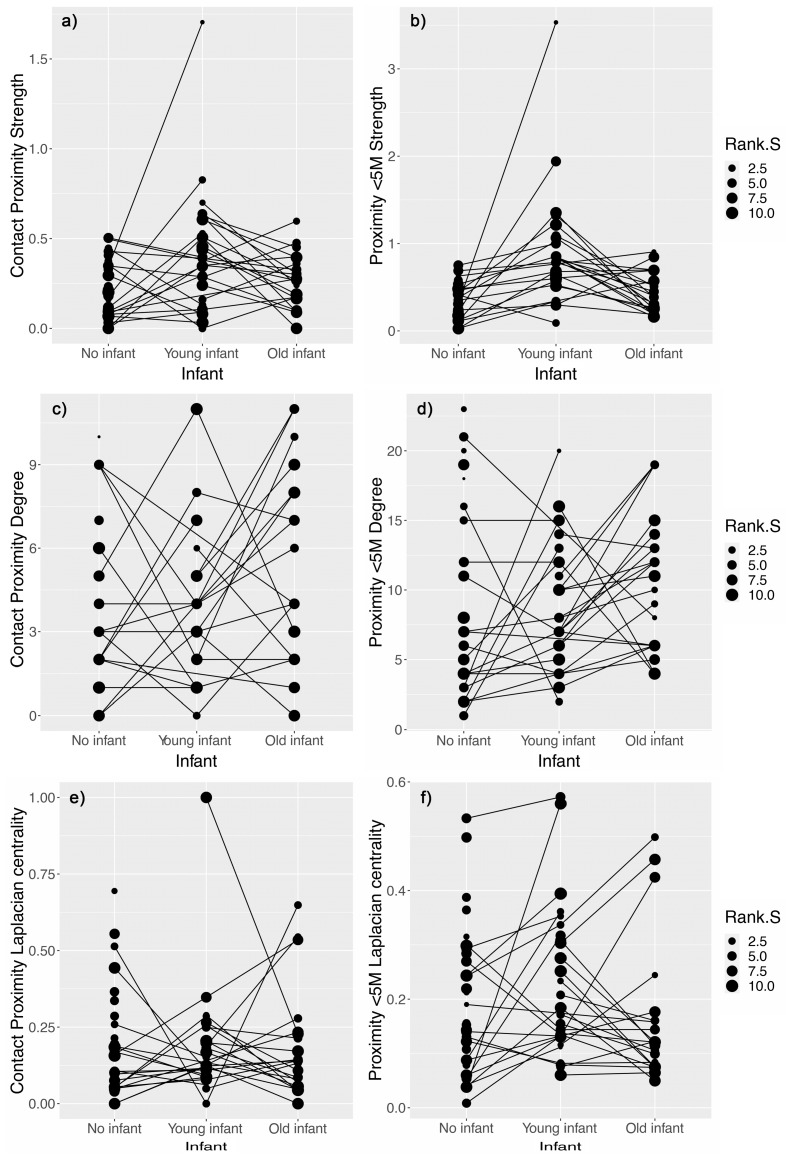
Comparison of the (**a**,**b**) frequency in association (*Strength*), (**c**,**d**) number of female neighbors (*Degree*) and (**e**,**f**) connectivity power (*Laplacian centrality*) between female nursing conditions (No infant (NN) vs. Young infant (YI) vs. Old infant (OI) females) of the contact- (**a**,**c**,**e**) and 5 m proximity networks (**b**,**d**,**f**). Node size reflects the hierarchical rank (the lowest values represent the highest-ranking individuals). The lines represent the same individual experiencing different nursing conditions in the course of the study. Because several individual data points could overlap, more than two lines could start from the same dot.

Partially in accordance with our predictions, we found that YI females were more frequently in contact (Figure 1a) and within 5 m of other females (Figure 1b) than OI and NN females, but had a lower number of contact partners compared to OI females (Figure 1c) (Table 1). Neither the number of neighbors (Figure 1d) in the 5 m-proximity network or the connectivity metric in both proximity networks (contact: Figure 1e; and 5 m: Figure 1f) differed significantly between the nursing conditions (Table 1). Whatever the proximity metrics tested, OI females did not significantly differ from NN females (Table 1).

**Figure 2:**
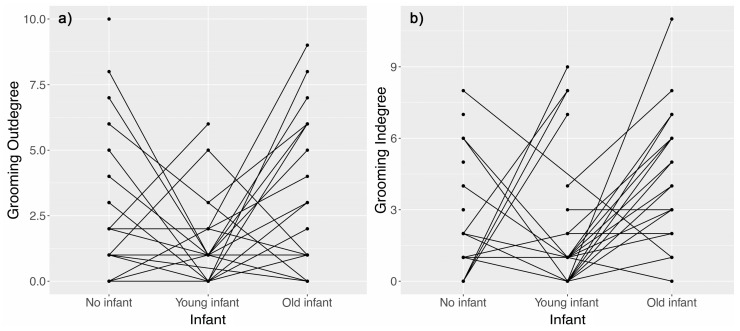
Comparison of the number of female partners (**a**) receiving (out-degree) or (**b**) giving (in-degree) grooming interactions across nursing condition (NN vs. YI vs. OI females). The lines represent the same individual experiencing different nursing conditions during the course of the study. Because several individual data points could overlap, more than two lines could start from the same dot.

For the grooming network, YI females were significantly less connected to the group than NN females, while not significantly different from OI females (Table 2). Contrary to the proximity network, we found no significant difference in grooming duration given and received between NN, YI and OI females. However, YI females groomed significantly fewer female partners than OI and NN females (Figure 2a). Similarly, YI females received grooming from a lower number of partners than OI females, but not so compared to NN females (Figure 2b). Whatever the grooming metrics tested, OI females did not significantly differ from NN females (Table 2).

Group size and hierarchical rank did not have any significant effect on the models of grooming network, whatever the SNA metric tested (Table 2). However, rank had an influence in proximity networks. The lower the hierarchical rank of a female (subordinate), the lower the frequency of time in contact with or at 5 m proximity from other females (Figure 1a,b, respectively), and the lower the number of female neighbors (Figure 1c,d, respectively) (Table 1). Finally, we found that the number of partners in 5 m proximity was smaller in Michelin (bigger) group than in Utara (smaller) group.

### 3.2. Effect of Sterilization on Grooming and Proximity Networks

**Figure 3:**
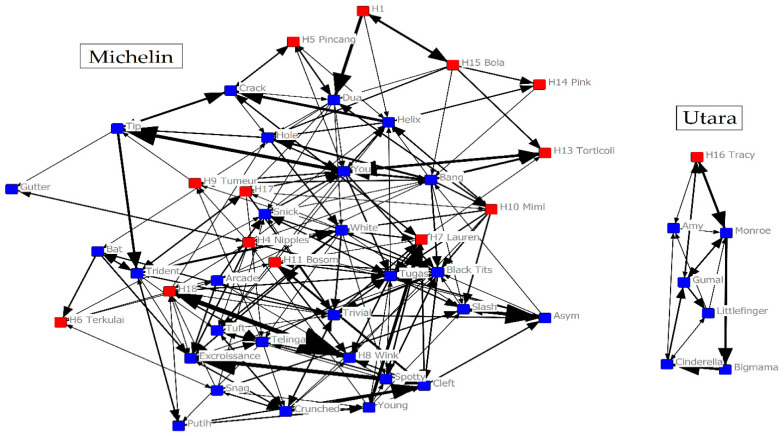
Grooming network of adult females in Michelin (**left**) and Utara (**right**) group at the Ubud Monkey Forest (early 2020). Color of nodes refers to the fertility status (red for sterilized females, blue for intact females). The arrows show the direction of the interactions and the tie size is scaled based on the strength of the relationship.

We controlled for the potential short-term effect of sterilization by testing the fertility status on grooming and proximity networks of the females. In the grooming network, the sterilized females did not significantly differ from intact females, whatever the SNA metric tested (Table 2). Thus, in accordance with our prediction, the social position of sterilized females did not significantly differ than intact females (Figure 3).

**Figure 4:**
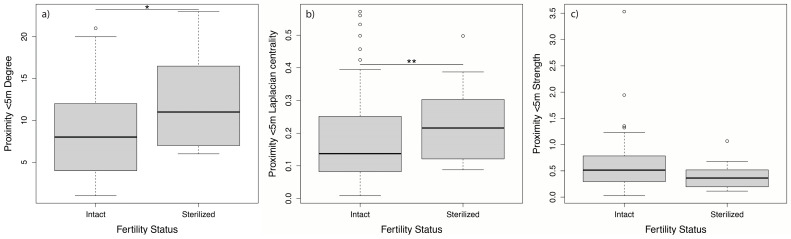
Comparison of the average (median) (**a**) Degree (number of female neighbors), (**b**) Laplacian centrality (connectivity power), and (**c**) Strength (frequencies of association) between sterilized and intact females for the 5 m proximity network (* *p* < 0.05, ** *p* < 0.01).

Unexpectedly, in the 5 m proximity network, sterilized females had significantly more neighbors (Figure 4a) and were better spatially connected (Figure 4b) than intact females, while no significant differences occurred in the contact-proximity network (Table 1). Despite the differences observed for the number of neighbors and the spatial connectedness role, we found no significant differences between sterilized and intact females in their frequency of associations in both proximity networks (Figure 4c).

## 4. Discussion

As sterilization is increasingly used to manage wild primate populations in anthropogenic environments [36,37,38,39,40,41,42,43], understanding if and how neutering may impact primate behavior and social organization is urgent. One of the first avenues to explore this relies on the role of unweaned infants in female sociality at the group level as a way to foresee whether the persisting absence of new offspring could hamper the quality of sterilized female social integration, and eventually impact the social cohesion and group stability likely to lead to group fission. In cercopithecine species, females with newborns are often the focus of other females’ attention [6,7,15,16]. To explore this question, we conducted a preliminary study in two groups of Balinese long-tailed macaques shortly after a sterilization program [43] to assess the general role played by the nursing condition and the potential short-term impact of sterilization (fertility status) on the female social position and role within their grooming and proximity networks. We found that YI females were more central (i.e., more frequently in contact/5 m) than OI and NN females in their spatial proximity networks. Conversely, when considering grooming networks, YI females were surprisingly less central (i.e., lower number of female partners) than OI and NN females and less connected in comparison to NN females. Regarding the fertility status, we found that sterilization did not have any positive or negative impact on female macaques’ social integration, at least in a short term.

### 4.1. Females with Young Unweaned Infants Were Less Central than Expected

YI females received grooming from fewer female partners than OI females and OI females showed centrality position similar to non-nursing females. These results are not consistent with a study on capuchin monkeys showing an increased number of grooming partners in lactating females [19]. Our results suppose that YI females could either display active avoidance [78,79] or were actually less attractive than expected [6,7,15,16,19]. As this population shows a non-seasonal reproduction [54], infants were present through the study period and at a substantial rate in this provisioned population. Consequently, we suppose that the large number of infants available makes them less attractive than when infants are scarce in social groups [11,80]. In parallel, YI females also initiated grooming to fewer partners than other females did while they did not significantly differ in grooming durations they emitted. As the number of partners is lower while the total grooming duration is similar, each grooming bout initiated per partner would last necessarily longer. These results are consistent with studies in chacma baboons (*P. ursinus*) where females promote the quality of grooming patterns through their degree of kinship and the strength of their social bonds (strong or weak) in order to adapt their social strategy and improve their individual fitness [81,82]. In our study, YI female macaques could select their partners (i.e., favor strong bonds) towards the most trustworthy females when approaching their newborn [83]. Therefore, we hypothesize that the matrilineal membership could have an effect on the number of favored grooming partners of nursing females, depending on the size and the dominance rank of the matriline, since grooming is preferentially directed towards kin-related and high-ranking females [3]. Moreover, Liao et al. [20] showed that older individual rhesus macaques (*M. mulatta*) selectively allocate social interactions to specific partners while younger individuals interact with a higher number of social partners. Consistently, adult female Japanese macaques (*M. fuscata*) with maternal experience were globally less interested by other females’ infants, which means they were less susceptible to interact with nursing females [15]. Thus, taking into account the age of the female subjects, their matriline belonging and their level of maternal experience in future analyses could bring a complementary explanation to our results.

Another possible explanation may be found in social style, including mother permissiveness. In many cercopithecine species, females are less permissive and tolerant during the first month of their infant life [13], infants being susceptible to be harassed, kidnapped or fatally injured, especially in despotic species as a form of reproductive competition [84,85] and in high density populations [84,86] where the social environment is more risky [86]. Although not despotic, *M. fascicularis* is a hierarchical species, classified as grade 2 on the 4-grade scale of macaque social style and characterized by a low degree of mother permissiveness [14]. Moreover, the population in Ubud Monkey Forest has for years experienced high density conditions and social tension [87,88]. It has been shown that, in captive macaques, crowding conditions increase infant harassment and kidnapping and lead to adjustments in parental style [86]. Kidnappings are quite common in the study population where we witnessed 43 events–including 3 deaths of infants–over 3 years of observation (unpublished data). In these conditions, YI females could actively avoid potential harassers [78,79,84] by limiting their grooming interactions to a low number of partners, probably mostly kin-related and trustworthy females, in order to reduce exposure of their young infant to harassment and kidnapping [83,86]. This could explain why females were more selective in their social interactions during the first months of their infant life [13] to become more central again with the development of the infant.

Finally, the lower centrality of YI females in grooming network could also be explained by time allocation differences between nursing conditions. While caring for a young infant may be time-consuming, old infants progressively become independent [56], leaving more time to their mother for grooming other females [89], just like non-nursing females do. As the average birth interval in our population is 1.2 years and weaning occurs at approximatively 12 months [56], the non-nursing condition was relatively short compared to the nursing one. These conditions could conceal the expected differences between non-nursing and nursing females. As for their potential role in the group connectivity (one index of group cohesion), non-nursing females showed higher connectivity power than YI females, which is consistent with their higher centrality in the grooming network.

Through spatial associations, the analysis of the proximity networks brought interesting results: YI females were more spatially central than OI and NN females by staying more often close (contact, 5 m) to other females, even though they did not significantly differ in their connectivity role in the group. Maintaining a central spatial position within the group for YI females promotes the protection of the young from environmental hazards, as suggested in other animal populations [90,91].

### 4.2. Sterilization Did neither Positively nor Neatively Impact the Female Social Networks in a Short Term

Taking into consideration the short time interval between the sterilizations and our study, and the life history variables of the study species (i.e., average time for an infant to be weaned and inter-birth interval), we predicted that sterilized females would not significantly differ from intact females in terms of individual centrality and group connectivity in both grooming and proximity networks. As the selected surgical technique (tubectomy) preserves the gonads and hormonal functions, we did not expect direct short-term effects on social behaviors [42,45,47]. In accordance with our prediction, we did not notice any significant difference between sterilized and intact females’ grooming (Figure 3) and contact-proximity networks. However, the question of the long-term impact of sterilization is still pending. As most females had been sterilized for less than one year at the beginning of the study (Appendix B), a period corresponding to the average inter-birth interval of the species [49], the short time interval did not allow to test the effect of long-term absence of new offspring, which could translate in a strong attraction of sterilized females towards other females’ infants [6,7,15,16]. Some sterilized females were even still caring an infant during a part of the study (Appendix B). Given the small sample size of females sterilized since more than one year (N = 2) in our study, a longer-term follow-up of sterilized females on a larger sample size is necessary to test the long-term effect of reproduction cessation on female social network.

The 5 m proximity network showed that sterilized females had significantly more female neighbors and a better group connectivity than intact females. Sexual activities may account for this result. As tubectomized females keep cycling without becoming gravid [42,45,47], they are susceptible to keep mating on longer periods [92], staying close to reproductive males, just like intact cycling females do [93,94,95]. Consequently, in a context of intra-sexual competition, they would keep close (5 m proximity) to other females, in an attempt to stay in the vicinity of males. Future research including male-female relationships are in progress to verify this sexual competition hypothesis for mate access.

## 5. Conclusions

The role of juvenile individuals is known to influence the group structure [21] and the social organization in primates [20]. By using SNA, this study provides the first evaluation of unweaned infant role on female macaque social network dynamics, and by extension, what this role might imply for sterilized females. Female macaques with young infants were not more central in the grooming network but enhanced their spatial position, as probably adapting their parental style and social interaction patterns to the high density conditions as similarly suggested by Maestripieri [86]. This study also verifies the absence of short-term implications of sterilization: tubectomy has no immediate negative consequence on female social position.

With regards to the limitations of our study, social dynamic data on a longer period would be necessary to deepen our understanding of the effect of reproduction cessation. It would be interesting to increase the time elapsed between the sterilizations and the behavioral observations to document the consequences of the permanent absence of new offspring and investigate whether the inter-individual difference in time since having the last offspring would dissimilarly impact the females’ social network metrics. Moreover, as the exclusion of specific age or sex classes from network analysis may lead to biased interpretation of network structure [21], including males and juveniles in future analyses could allow to reach a more refined understanding of the infant role in the entire group dynamics. In complement, the measure of hormonal correlates of the ovarian cycle would allow to clarify the reproductive conditions of the non-nursing females and their influence on social behaviors to further identify the social importance of having or not an unweaned infant. Finally, the role of sexual competition [93,96] through changes in the operational sex ratio following birth control might have its importance as well and should be investigated in future studies.

Compiling further data on sterilization in primates has not only a fundamental interest to enhance our knowledge about animal social dynamics, but also a major applied interest to promote the most informed decisions and welfare-focused choices in management strategies of captive and wild primate populations. Social network analysis helps to assess group stability and changes following management decision [31,97]. Social instability detection may prevent social fragmentation or improve suitability of management actions. Combining the use of SNA with systematic welfare measures might help to anticipate and assess the consequences of human interventions [97,98]. Further studies should investigate correlation between welfare behavioral and physiological indicators and network measures of social cohesion and stability in the framework of reproduction control to identify structural dynamics increasing welfare [99]. Given the paucity of information currently available in this realm, we call for systematic monitoring and investigation of the sterilization-related questions by taking advantage of existing birth control programs in the wild and captive settings.

## Figures and Tables

**Table 1:** Proximity network. Generalized linear mixed models (mixed binomial (degree) or gaussian (strength and Laplacian centrality) regressions) of SNA proximity (contact or ≤5 m) metrics, testing the main fixed effects of nursing condition (YI vs. OI vs. NN females), and the controlled predictors (i.e., fertility status (intact vs. sterilized females), group size and hierarchical rank): coefficient estimates, standard errors, *z*-test (for degree), or *t*-test (for strength and Laplacian centrality) and *p*-values, and likelihood ratio tests (LRT). The reference category for the nursing condition is the first condition stated. ^a^ “Intact females” as a reference category for the fertility status. The lowest values of the hierarchical rank represent the highest-ranked individuals.

Response Variable	Proximity	Fixed Effects	Estimate ± SE	*z*/*t* Values	*p*-Values
Degree	Contact	Intercept	−0.41 ± 0.33	−1.23	0.11
		Nursing condition			
		YI-NN	0.06 ± 0.15	0.42	0.73
		YI-OI	0.39 ± 0.14	2.75	<0.05
		OI-NN	−0.33 ± 0.15	−2.19	0.11
		Status ^a^	0.08 ± 0.17	0.47	0.69
		Rank	−0.07 ± 0.02	−3.04	<0.01
		Group size	−0.03 ± 0.01	−4.24	0.11
		LRT: deviance = 396.74, Df = 5, *p* < 0.0001			
	≤5 m	Intercept	1.62 ± 0.37	4.39	<0.05
		Nursing condition			
		YI-NN	−0.07 ± 0.13	−0.53	0.76
		YI-OI	0.28 ± 0.12	2.42	0.23
		OI-NN	−0.35 ± 0.14	−2.51	0.16
		Status ^a^	0.45 ± 0.18	2.52	<0.05
		Rank	−0.09 ± 0.03	−3.50	<0.01
		Group size	−0.06 ± 0.01	−6.94	<0.05
		LRT: deviance = 518.75, Df = 5, *p* < 0.0001			
Strength	Contact	Intercept	0.35 ± 0.09	4.01	<0.001
		Nursing condition			
		YI-NN	−0.20 ± 0.06	−3.66	<0.001
		YI-OI	−0.15 ± 0.06	−2.59	<0.05
		OI-NN	0.06 ± 0.06	−0.91	0.07
		Status ^a^	−0.03 ± 0.06	−0.49	0.72
		Rank	−0.02 ± 0.01	−3.10	<0.01
		Group size	0.006 ± 0.002	3.18	0.32
		LRT: deviance = −37.24, Df = 5, *p* < 0.0001			
	≤5 m	Intercept	1.15 ± 0.09	12.42	<0.001
		Nursing condition			
		YI-NN	−0.37 ± 0.06	−6.32	<0.001
		YI-OI	−0.28 ± 0.06	−4.53	<0.001
		OI-NN	−0.09 ± NA	NA	0.25
		Status ^a^	0.05 ± 0.07	0.71	0.58
		Rank	−0.02 ± 0.01	−3.03	<0.05
		Group size	−0.003 ± 0.002	−1.59	0.16
		LRT: deviance = −28.22, Df = 5, *p* < 0.0001			
Laplacian centrality	Contact	Intercept	0.64 ± 0.08	8.53	0.15
		Nursing condition			
		YI-NN	−0.051 ± 0.047	−1.09	0.28
		YI-OI	−0.01 ± 0.05	−0.14	0.90
		OI-NN	−0.04 ± 0.05	−0.91	0.37
		Status ^a^	0.052 ± 0.054	0.98	0.29
		Rank	−0.009 ± 0.007	−1.27	0.21
		Group size	−0.006 ± 0.002	−3.75	0.19
		LRT: deviance = −62.93, Df = 5, *p* < 0.05			
	≤5 m	Intercept	0.43 ± 0.05	8.86	0.27
		Nursing condition			
		YI-NN	−0.06 ± 0.03	−2.06	0.053
		YI-OI	−0.05 ± 0.03	−1.79	0.09
		OI-NN	−0.01 ± 0.03	−0.17	0.87
		Status ^a^	0.10 ± 0.03	2.82	<0.01
		Rank	−0.0004 ± 0.004	−0.10	0.93
		Group size	−0.006 ± 0.001	−6.07	0.07
		LRT: deviance = −141.96, Df = 5, *p* < 0.0001			

**Table 2:** Grooming network. Generalized linear mixed models (mixed binomial (in-/out-degree) or gaussian (in-/out-strength and Laplacian centrality) regressions) of SNA grooming metrics, testing the main fixed effects of nursing condition (YI vs. OI vs. NN females), and the controlled predictors (i.e., fertility status (intact vs. sterilized females), group size and hierarchical rank): coefficient estimates, standard errors, *z*-test (for in-/out-degree) or *t*-test (for in-/out-strength and Laplacian centrality) and *p*-values, and likelihood ratio tests (LRT). The reference category for the nursing condition is the first condition stated. ^a^ “Intact females” as a reference category for the fertility status. The lowest values of the hierarchical rank represent the highest-ranked individuals.

Response Variable	Fixed Effects	Estimate ± SE	*z*/*t* Values	*p* Values
In-degree	Intercept	−1.00 ± 0.40	−2.51	0.81
	Nursing condition			
	YI-NN	0.39 ± 0.20	2.00	0.19
	YI-OI	0.89 ± 0.19	4.69	<0.01
	OI-NN	−0.49 ± 0.19	−2.57	0.13
	Status ^a^	0.05 ± 0.22	0.25	0.87
	Rank	−0.07 ± 0.03	−2.40	0.10
	Group size	−0.04 ± 0.01	−4.52	0.43
	LRT: deviance = 377.22, Df = 5, *p* < 0.0001			
Out-degree	Intercept	−1.47 ± 0.43	−3.42	0.21
	Nursing condition			
	YI-NN	0.92 ± 0.21	4.40	<0.01
	YI-OI	1.02 ± 0.21	4.92	<0.001
	OI-NN	−0.10 ± 0.19	−0.50	0.79
	Status ^a^	0.23 ± 0.22	1.04	0.52
	Rank	−0.05 ± 0.03	−1.42	0.33
	Group size	−0.04 ± 0.01	−4.23	0.36
	LRT: deviance = 349.58, Df = 5, *p* < 0.0001			
In-strength	Intercept	0.012 ± 0.013	0.93	0.36
	Nursing condition			
	YI-NN	0.004 ± 0.01	0.39	0.71
	YI-OI	0.02 ± 0.01	1.91	0.07
	OI-NN	−0.02 ± 0.01	−1.49	0.14
	Status ^a^	−0.013 ± 0.011	−1.15	0.26
	Group size	0.0005 ± 0.0003	1.84	0.51
	LRT: deviance = −325.93, Df = 4, *p* = 0.06			
Out-strength	Intercept	0.03 ± 0.02	1.56	0.98
	Nursing condition			
	YI-NN	0.015 ± 0.010	1.48	0.16
	YI-OI	0.0101 ± 0.0102	1.00	0.34
	OI-NN	0.004 ± 0.01	0.40	0.70
	Status ^a^	−0.008 ± 0.01	−0.66	0.50
	Rank	−0.001 ± 0.002	−0.82	0.40
	Group size	0.00032 ± 0.00035	0.94	0.92
	LRT: deviance = −325.66, Df = 5, *p* = 0.49			
Laplacian centrality	Intercept	0.56 ± 0.15	3.82	0.20
	Nursing condition			
	YI-NN	0.19 ± 0.08	2.27	<0.05
	YI-OI	0.10 ± 0.09	1.11	0.29
	OI-NN	0.091 ± 0.093	0.98	0.34
	Status ^a^	−0.04 ± 0.10	−0.39	0.70
	Rank	0.002 ± 0.01	0.17	0.88
	Group size	−0.002 ± 0.003	−0.60	0.56
	LRT: deviance = 29.76, Df = 5, *p* = 0.29			

## Data Availability

Not applicable.

## Appendix A

**Table A1:** Demographic status of the macaque population and composition of the two study groups (Michelin and Utara) in Ubud Monkey Forest in March 2020 (i.e., at the end of the study period); with number of studied intact and sterilized females.

**Population**	**Ubud**	
Number of social groups	8	
Population size	1099	
Home range size (ha)	20.5	
Density (ind/ha)	54	
**Study groups**	**Michelin**	**Utara**
Group size	136	33
Adult males	16	7
Adult females	45	9
Subadult males	12	3
Subadult females	5	2
Juveniles	43	8
Old infant	8	3
Young infant	7	1
**Total females studied**	**41**	**7**
Sterilized females	13	1
Intact females	28	6

## Appendix B

**Table A2:** Summary of the focal subjects, with for each of them: group, age class (adult female vs. subadult female), standardized hierarchical rank, fertility status (intact vs. sterilized), time (in months) elapsed (by the middle of the study) since sterilization surgery, and the nursing conditions (nursing a young infant (YI), nursing an old infant (OI), non-nursing females) experienced during the study period.

						Nursing Condition		
ID	Group	Age	Rank	Status	Time	Nursing YI	Nursing OI	Non-Nursing
Monroe	Utara	AF	1.43	Intact	-	No	No	Yes
Gumal	Utara	AF	2.86	Intact	-	Yes	Yes	Yes
H16 Tracy	Utara	AF	4.29	Sterilized	4	No	No	Yes
Littlefinger	Utara	AF	5.71	Intact	-	Yes	No	Yes
Cinderella	Utara	AF	7.14	Intact	-	Yes	Yes	Yes
Bigmama	Utara	AF	8.57	Intact	-	Yes	Yes	No
Amy	Utara	AF	10	Intact	-	Yes	No	Yes
H4 Nipples	Michelin	AF	0.24	Sterilized	10	No	No	Yes
Snick	Michelin	AF	0.49	Intact	-	Yes	No	Yes
Cleft	Michelin	AF	0.73	Intact	-	No	Yes	Yes
Asym	Michelin	AF	0.98	Intact	-	No	No	Yes
H11 Bosom	Michelin	AF	1.22	Sterilized	10	No	No	Yes
Young	Michelin	AF	1.46	Intact	-	Yes	Yes	No
Putih	Michelin	AF	1.71	Intact	-	Yes	Yes	No
Bat	Michelin	AF	1.95	Intact	-	Yes	No	Yes
You	Michelin	AF	2.2	Intact	-	Yes	Yes	No
BlackTits	Michelin	AF	2.44	Intact	-	Yes	No	Yes
Crunched	Michelin	AF	2.68	Intact	-	Yes	No	Yes
Spotty	Michelin	AF	2.93	Intact	-	No	No	Yes
Telinga	Michelin	AF	3.17	Intact	-	Yes	Yes	No
H7 Lauren	Michelin	AF	3.41	Sterilized	10	No	No	Yes
H8 Wink	Michelin	AF	3.66	Intact	-	Yes	Yes	No
Trivial	Michelin	AF	3.9	Intact	-	Yes	Yes	No
Excroissance	Michelin	AF	4.15	Intact	-	Yes	Yes	No
H13 Torticoli	Michelin	AF	4.39	Sterilized	4	No	No	Yes
Slash	Michelin	AF	4.63	Intact	-	Yes	No	Yes
H18	Michelin	SF	4.88	Sterilized	4	No	No	Yes
Tip	Michelin	AF	5.12	Intact	-	No	No	Yes
White	Michelin	AF	5.37	Intact	-	Yes	Yes	Yes
Tugas	Michelin	AF	5.61	Intact	-	Yes	Yes	No
Tuft	Michelin	AF	5.85	Intact	-	Yes	Yes	Yes
Trident	Michelin	AF	6.1	Intact	-	Yes	No	Yes
H5 Pincang	Michelin	AF	6.34	Sterilized	17	No	No	Yes
H1	Michelin	AF	6.59	Sterilized	29	No	No	Yes
H6 Terkulai	Michelin	AF	6.83	Sterilized	10	No	No	Yes
Arcade	Michelin	AF	7.07	Intact	-	Yes	Yes	Yes
H10 Mimi	Michelin	AF	7.32	Sterilized	10	Yes	Yes	No
H9 Tumeur	Michelin	AF	7.56	Sterilized	10	No	No	Yes
Snag	Michelin	AF	7.8	Intact	-	No	No	Yes
H17	Michelin	SF	8.05	Sterilized	4	No	No	Yes
H14 Pink	Michelin	AF	8.29	Sterilized	4	No	Yes	Yes
Helix	Michelin	AF	8.54	Intact	-	Yes	No	Yes
Gutter	Michelin	AF	8.78	Intact	-	Yes	Yes	Yes
Crack	Michelin	AF	9.02	Intact	-	Yes	No	Yes
Bang	Michelin	AF	9.27	Intact	-	Yes	Yes	No
Hole	Michelin	AF	9.51	Intact	-	Yes	Yes	No
Dua	Michelin	AF	9.76	Intact	-	Yes	Yes	No
H15	Michelin	SF	10	Sterilized	4	No	No	Yes
